# Progress in adjuvant chemotherapy for breast cancer: an overview

**DOI:** 10.1186/s12916-015-0439-8

**Published:** 2015-08-17

**Authors:** Jesus Anampa, Della Makower, Joseph A. Sparano

**Affiliations:** Department of Oncology, Montefiore Medical Center, Albert Einstein College of Medicine, Albert Einstein Cancer Center, Bronx, NY 10461 USA

**Keywords:** Adjuvant chemotherapy, Anthracyclines, Breast cancer, Chemotherapy, Early breast cancer, Taxanes

## Abstract

Breast cancer is the most common cause of cancer and cancer death worldwide. Although most patients present with localized breast cancer and may be rendered disease-free with local therapy, distant recurrence is common and is the primary cause of death from the disease. Adjuvant systemic therapies are effective in reducing the risk of distant and local recurrence, including endocrine therapy, anti-HER2 therapy, and chemotherapy, even in patients at low risk of recurrence. The widespread use of adjuvant systemic therapy has contributed to reduced breast cancer mortality rates. Adjuvant cytotoxic chemotherapy regimens have evolved from single alkylating agents to polychemotherapy regimens incorporating anthracyclines and/or taxanes. This review summarizes key milestones in the evolution of adjuvant systemic therapy in general, and adjuvant chemotherapy in particular. Although adjuvant treatments are routinely guided by predictive factors for endocrine therapy (hormone receptor expression) and anti-HER2 therapy (HER2 overexpression), predicting benefit from chemotherapy has been more challenging. Randomized studies are now in progress utilizing multiparameter gene expression assays that may more accurately select patients most likely to benefit from adjuvant chemotherapy.

## Background

Breast cancer is the most frequently diagnosed cancer and the leading cause of cancer death among women, accounting for 25 % of the total cancer cases (1.68 million) and 15 % of the cancer deaths (520,000) worldwide [1, 2]. In the United States, it is estimated that there will be 231,840 new cases of invasive breast cancer and 40,290 deaths from the disease in 2015, and that one in eight women will develop breast cancer during their lifetime [3]. The disease is localized to the breast at presentation in 61 % of cases, regionally advanced in 32 %, and metastatic in 7 % [4]. When localized or regionally advanced, the disease is potentially curable with local and systemic therapy. Adjuvant systemic therapies reduce the risk of distant recurrence, presumably by treating micro-metastatic disease that may not be clinically evident at the time of local therapy. Prognostic factors for distant recurrence irrespective of treatment include classical clinicopathologic features such as tumor size, tumor grade, and number of axillary lymph nodes with metastasis. Predictive factors that identify benefit from specific therapies include expression of the estrogen receptor (ER) and progesterone receptor (PR), which identify patients who benefit from adjuvant endocrine therapy [5], and overexpression of human epidermal growth factor receptor 2 (HER2) protein (or HER2 gene amplification) [6], which identifies patients who benefit from adjuvant HER2-directed therapy. Multiparameter gene expression assays may also provide both prognostic information and prediction of benefit from adjuvant chemotherapy in patients with ER-positive disease [7, 8].

### An abbreviated history of adjuvant systemic therapy

The initial approach to therapy for breast cancer was based on the premise that the disease metastasized via locoregional spread in an orderly fashion, and thus could be cured with aggressive surgery. The radical mastectomy was thus the standard surgical procedure for breast cancer in the early 20^th^ century [9]. Randomized trials subsequently showed no benefit from radical mastectomy compared with less aggressive surgical procedures, and demonstrated that distant recurrence remained a major clinical problem irrespective of the primary surgical therapy [10, 11].

As the approach to local therapy evolved from more aggressive to less aggressive, the types of adjuvant systemic therapies and their indications expanded. A series of seminal clinical trials demonstrated that adjuvant systemic chemotherapy, endocrine therapy, and anti-HER2 directed therapy substantially reduced the risk of recurrence and improved overall survival when added to local therapy. In addition to the milestones achieved by individual trials summarized in Box 1, the Early Breast Cancer Trialists’ Collaborative Group (EBCTCG) has periodically reported meta-analyses of all clinical trials with available data that have added to our knowledge about the benefits of adjuvant systemic therapy [12–16]. Based upon the improvements in outcomes associated with systemic therapies described below, current adjuvant therapy options summarized in Table 1 are commonly tailored to four phenotypic subtypes that are defined in a practical manner by utilizing information on ER, PR, and HER2 expression. This practical phenotypic classification roughly corresponds to “intrinsic subtypes” identified by gene expression profiling [17], although the latter classification may provide more accurate prognostic and predictive information [18, 19].

**Table 1:** Systemic adjuvant therapy options for operable breast cancer

Breast cancer subtype/classification			Adjuvant systemic therapy		
Phenotypic subtype		Intrinsic subtype	Endocrine therapy	Anti-HER2 therapy	Chemotherapy
Hormone receptors	HER2 overexpression				
**+**	**–**	Luminal A or B	Yes	No	Yes (if high risk)
**+**	**+**	Luminal B or HER2 enriched	Yes	Yes	Yes
**–**	**–**	Basal	No	No	Yes
**–**	**+**	HER2 enriched	No	Yes	Yes

The first randomized trial evaluating adjuvant chemotherapy in breast cancer was the National Surgical Adjuvant Breast and Bowel Project (NSABP) B-01 trial initiated in 1958, which reported in 1968 that an adjuvant alkylating agent (thiotepa) given after radical mastectomy significantly decreased recurrence rate in pre-menopausal women with four or more positive axillary lymph nodes [20]. A subsequent randomized study reported in 1975 showed benefit from another alkylating agent (L-phenylalinine mustard) [21]. Other reports from the Istituto Nazionale Tumori in Milan, Italy, showed that combination chemotherapy regimen called “CMF” including an alkylating agent (cyclophosphamide) and antimetabolites (methotrexate and 5-fluorouracil) significantly reduced the risk of recurrence [22], thus ushering in the modern age of adjuvant polychemotherapy regimens that are now commonly used in clinical practice. These trials were among the first to establish a role for adjuvant chemotherapy, initially in premenopausal women with axillary node-positive disease at highest risk for recurrence [22], with subsequent trials also showing benefit in lower risk post-menopausal women [23] and women with axillary node-negative disease [24–26]. In 2001, a National Institute of Health consensus panel in the US concluded: “*Because adjuvant polychemotherapy improves survival, it should be recommended to the majority of women with localized breast cancer regardless of lymph node, menopausal, or hormone receptor status*.” [27] Although the widespread adoption of more effective systemic therapies contributed to declining breast cancer mortality rates in the US and globally [1, 28], it also resulted in many patients being unintentionally “overtreated” with chemotherapy who might otherwise may been cured without it. Several multiparameter gene expression assays have recently been shown to provide prognostic information in patients with ER-positive breast cancer [7, 8] and also identify which patients derive greatest benefit from adjuvant chemotherapy [29, 30]. Some of these assays are endorsed by evidence-based guidelines for making clinical decisions regarding the use of adjuvant chemotherapy in specific settings [31].

Approximately 75 % of all breast cancers express hormone receptors [5]. Endocrine therapy reduces the risk of recurrence in hormone receptor-expressing disease, whether used alone or in addition to chemotherapy. In 1982, adjuvant tamoxifen given for 2 years was shown to reduce the risk of recurrence [32] and improve survival [33]. Subsequent studies and a meta-analyses of these studies confirmed a survival benefit [12], and also showed that 5 years of therapy was more effective than shorter durations, the proportional benefits were similar irrespective of nodal metastasis, and that the benefits were seen only in patients with ER-positive tumors [13, 15]. Aromatase inhibitors were subsequently shown to be more effective than tamoxifen in postmenopausal women [34, 35]. In addition, extended adjuvant therapy for up to 10 years was shown to be more effective than 5 years of therapy, including sequential tamoxifen followed by an aromatase inhibitor [36], or tamoxifen for up to 10 years [37]. Finally, in premenopausal women at high risk for recurrence, ovarian suppression plus an aromatase inhibitor was shown to be more effective than tamoxifen [38, 39].

Approximately 25 % of all breast cancers overexpress the HER2 oncogene [6]. In 2005, several randomized trials demonstrated that addition of the anti-HER2 antibody trastuzumab to adjuvant chemotherapy, either concurrently or sequentially, substantially decreased the risk of recurrence in patients with HER2 overexpressing node-positive or high-risk node-negative breast cancer [40–43]. Addition of trastuzumab to sequential anthracycline/cyclophosphamide-taxane was associated with about a 3 % risk of cardiac toxicity [40–42], while the combination of trastuzumab with non-anthracycline regimens (e.g. carboplatin/docetaxel) was associated with lower rates of cardiac toxicity [43]. Non-randomized single arm studies have also shown excellent outcomes in patients with lower risk node-negative disease not included in other studies who would have been expected to have higher recurrence rates without adjuvant trastuzumab [44, 45]. Subsequent studies demonstrated that 1 year of trastuzumab was more effective than 6 months [46], but 2 years of therapy was no more effective than 1 year [47]. The addition of the HER2 tyrosine kinase inhibitor lapatinib did not improve outcomes when added to trastuzumab [48].

### Adjuvant chemotherapy: first, second, and third generation regimens

Adjuvant! is a web-based decision aid commonly used in clinical practice that allows clinicians and patients to better understand the potential benefits of adjuvant therapy, especially chemotherapy [49]. Estimates provided by Adjuvant! have been shown to correlate closely with actual clinical outcomes in population- and hospital-based cohorts [50, 51]. Adjuvant! classifies adjuvant chemotherapy regimens as first, second, and third-generation, as exemplified in Table 2. A modification of this classification will be used here to categorize the numerous chemotherapy regimens discussed in this review, and to describe the clinical trials summarized in Table 3. The regimens used in these studies generally included anthracyclines (doxorubicin, epirubicin) and/or taxanes (paclitaxel, docetaxel), which are the two most active classes of cytotoxic agents for both early and advanced stage breast cancer.

**Table 2:** Classification of adjuvant chemotherapy regimens

Generation*	Benefit	Regimens with substantial evidence base
First	35 % reduction in breast cancer mortality compared with no adjuvant chemotherapy	CMFx6, ACx4, FEC50x6
Second	20 % reduction in breast cancer mortality compared with first generation regimen	FEC100x6, CAFx6, FACx6
		ACx4-Tx4 (q3wks)
		DCx4, Ex4-CMFx4
Third	20 % reduction in breast cancer mortality compared with second generation regimen	FECx4-Dx3, FECx4-weekly Tx8
		Concurrent DAC
		Dose-dense ACx4-Tx4
		ACx4-weekly paclitaxel
		ACx4-docetaxel (q 3 weeks)

*Adopted from Adjuvant online with modifications [50, 51]

CMF, Cyclophosphamide, methotrexate, 5-flourouracil; AC, Doxorubicin, cyclophosphamide; FEC50, 5-flourouracil, epirubicin (50 mg/m^2^), cyclophosphamide; FEC100, 5-flourouracil, epirubicin (100 mg/m^2^), cyclophosphamide; DC, Docetaxel, cyclophosphamide; CAF, Cyclophosphamide, doxorubicin, 5-flourouracil; FAC, 5-flouroracil, doxorubicin, cyclophosphamide; DAC, Docetaxel, doxorubicin, cyclophosphamide; T, paclitaxel; D, Docetaxel; E, Epirubicin

**Table 3:** Select phase III trials of first, second, and third generation trials

Generation	Comparison (Reference)	Nodal status	Number of patients	Median follow-up (years)	Hazard ratio for disease-free survival	Hazard ratio for overall survival
First	CMF vs no chemo [22, 71]	Positive	386	28.5	0.71 (*P* = 0.005)	0.79 (*P* = 0.04)
	CMF + Tam vs Tam (B20) [26]	Negative	2306	5	0.65 (*P* = 0.001)	0.64 (*P* = 0.03)
	AC vs CMF (B15) [72, 73]	Positive	2194	3	*P* = 0.5*	*P* = 0.8*
	AC vs CMF (B23) [74]	Negative	2008	5	*P* = 0.9*	*P* = 0.4*
	FEC50 + Tam vs Tam [76]	Positive	457	9.4	0.46 (*P* = 0.0008)	0.65 (*P* = 0.07)
Second	FEC100 vs FEC50 [78]	Positive	546	5.6	0.63 (*P* = 0.02)	0.45 (*P* = 0.005)
	ACx4-Tx4 vs ACx4 (C9344) [82]	Positive	3121	5.8	0.83 (*P* = 0.002)	0.82 (*P* = 0.006)
	ACx4-Tx4 vs ACx4 (B28) [83]	Positive	3060	5.4	0.83 (*P* = 0.006)	0.93 (*P* = 0.46)
	DCx4 vs ACx4 [85]	0–3 Positive	1016	7	0.74 (*P* = 0.033)	0.69 (*P* = 0.032)
	Ex4-CMFx4 vs CMFx6/CMFx8 [84]	Positive Negative	2391	4	0.69 (*P* <0.001)	0.67 (*P* <0.001)
Third	DAC vs FAC [89, 112]	Positive	1491	10.3	0.80 (*P* = 0.004)	0.74 (*P* = 0.002)
	DAC vs FAC [91]	Negative	1060	6.4	0.68 (*P* = 0.01)	0.76 (*P* = 0.29)
	FEC-D vs FEC [92, 93]	Positive	1099	7.8	0.85 (*P* = 0.036)	0.75 (*P* = 0.007)
	FEC-weekly T vs FEC [95]	Positive	1246	5.5	0.77 (*P* = 0.022)	0.78 (*P* = 0.11)
	FAC-weekly T vs FAC [113]	Negative	1925	5.3	0.73 (*P* = 0.04)	0.79 (*P* = 0.31)
	Q3 vs q2wk ACT [97, 98]	Positive	2005	5.8	0.80 (*P* = 0.01)	0.85 (*P* = 0.04)
	AC-T vs AC-weekly T [100, 101]	Positive	4954	12.1	0.84 (*P* = 0.011)	0.87 (*P* = 0.09)
	AC-T vs AC-D				0.79 (*P* = 0.001	0.86 (*P* = 0.054)
	AC-D vs DAC [103]	Positive	5351	6.1	0.83 (*P* = 0.01)	0.86 (*P* = 0.09)
	AC-D vs AD				0.80 (*P* = 0.001)	0.83 (*P* = 0.03)

CMF, Cyclophosphamide, methotrexate, 5-flourouracil; AC, Doxorubicin, cyclophosphamide; FEC50, 5-flourouracil, epirubicin (50 mg/m^2^), cyclophosphamide; FEC100, 5-flourouracil, epirubicin (100 mg/m^2^), cyclophosphamide; DC, Docetaxel, cyclophosphamide; CAF, Cyclophosphamide, doxorubicin, 5-flourouracil; FAC, 5-flouroracil, doxorubicin, cyclophosphamide; DAC, Docetaxel, doxorubicin, cyclophosphamide; T, Paclitaxel; D, Docetaxel; E, Epirubicin

*Hazard ratios were not reported in the manuscript; however *P* values did not reveal any statistical significance between study arms

#### Anthracyclines

Anthracyclines, derivatives of the antibiotic rhodomycin B, were initially isolated in the 1950s from gram-positive Streptomyces present in an Indian soil sample [52]. Doxorubicin was isolated from *Streptomyces peucetius* [53], a mutant of the original Streptomyces strain found near the Adriatic sea, and was therefore named Adriamycin. Doxorubicin was found to be one of the most active single cytotoxic agents in metastatic breast cancer [54, 55], although congestive cardiomyopathy emerged as a toxicity that required limiting the cumulative lifetime dose in order to minimize the risk of this toxicity [56]. Epirubicin, an epimer of doxorubicin differing in the orientation of the C4 hydroxyl group on the sugar, is a less cardiotoxic anthracycline than doxorubicin [57, 58].

#### Taxanes

Paclitaxel was originally isolated from the bark of the Pacific yew tree *taxus brevifolia*, and its antitumor activity was initially described in 1971 [59]. Paclitaxel binds to microtubules and induces their stabilization by inhibiting their depolymerization, thereby leading to mitotic arrest [60, 61] and chromosome missegregation on abnormal multipolar spindles [62, 63]. Despite its unique mechanism of action, paclitaxel’s initial development was slow due to its scarcity and poor solubility. A formulation of paclitaxel solubilized in Cremophor EL was eventually developed but was associated with hypersensitivity reactions to the Cremophor EL vehicle [64], requiring premedication with corticosteroids and histamine blockers, which nearly thwarted paclitaxel’s clinical development. In 1994, Cremophor-EL-paclitaxel was approved by the United States Food and Drug Administration (FDA) for treatment of metastatic breast cancer in patients who had progressed after anthracycline-based combination chemotherapy or who relapsed less than 6 months after adjuvant therapy [64]. In order to address the initial scarcity of paclitaxel, docetaxel, a semi-synthetic agent derived from the needles of the European yew tree *taxus baccata*, was developed [65]. Docetaxel has a similar mechanism of action to paclitaxel, but is a more potent microtubule inhibitor *in vitro* [65]. Docetaxel is also slightly more water-soluble than paclitaxel, and is dissolved in polysorbate-80. Despite the different solvent, premedication is also required to reduce the risk of acute hypersensitivity reactions and cumulative fluid retention associated with docetaxel infusions [66]. A direct comparison of docetaxel with paclitaxel in metastatic breast cancer showed greater efficacy for docetaxel but more toxicity [67], whereas a direct comparison of paclitaxel with doxorubicin as first line therapy showed comparable efficacy [68]. Both of these agents have been extensively tested in adjuvant trials based upon substantial single agent activity for each agent in metastatic breast cancer [69].

### First-generation chemotherapy regimen

#### Cyclophosphamide, methotrexate, and 5-fluorouracil (CMF)

CMF was the first combination adjuvant chemotherapy regimen that was tested in a prospective clinical trial (Table 3). This trial, initiated in 1973 by the Istituto Nazionale Tumori in Milan, Italy, randomized node positive patients after radical mastectomy to 12 cycles of cyclophosphamide (100 mg/m^2^ orally on days 1–14), methotrexate (40 mg/m^2^ IV on days 1 and 8), and 5-fluorouracil (600 mg/m^2^ IV on days 1 and 8) administered every 28 days versus no additional treatment [22]. The updated 342-month follow-up reported an improved disease-free survival (DFS; hazard ratio (HR), 0.71; *P* = 0.005) and overall survival (OS; HR, 0.79; *P* = 0.04) for CMF compared with the control population. Another important finding was that relapse rate was no different when pre-menopausal women were compared to post-menopausal women. A subsequent study demonstrated that six cycles was as effective 12 cycles of adjuvant CMF [70], with results sustained after long-term follow-up [71]. A study conducted by the US Breast Cancer Intergroup found that six cycles of adjuvant CMF was also effective in reducing the risk of recurrence and improving survival in axillary node-negative disease [24, 25]. For patients with ER-positive, lymph node-negative disease at lower risk for recurrence, the NSABP B-20 trial found that the addition of CMF to tamoxifen improved 5-year DFS (HR, 0.65; *P* = 0.001) and OS (HR, 0.64; *P* = 0.03) [26]. The EBCTCG meta-analysis found that adjuvant CMF reduced the risk of recurrence by 30 % at 10 years (HR, 0.70; *P* <0.00001), which translated into an absolute gain of 10.2 %. The 10-year overall mortality risk was reduced by 16 % (HR, 0.84; *P* <0.0004), which translated into an absolute gain of 4.7 % at 10 years [16].

#### Doxorubicin and cyclophosphamide (AC)

One of the first adjuvant trials evaluating doxorubicin was NSABP B-11, which compared melphalan and 5-fluorouracil with or without doxorubicin in 697 non-tamoxifen responsive patients (defined as women 50–59 years with a tumor PR level by ligand binding assay of 0–9 fmol, and all patients ≤49 years), and found an improved 5-year DFS (HR, 0.65; *P* = 0.007) and a trend to improved OS (HR, 0.74; *P* = 0.08) for the doxorubicin arm [72]. In order to find a more intense and shorter chemotherapy regimen, the NSABP B-15 trial randomized 2,194 patients with node-positive disease to AC (doxorubicin 60 mg/m^2^ and cyclophosphamide 600 mg/m^2^ every 3 weeks for four cycles) given over 12 weeks versus conventional CMF for six cycles over 24 weeks (Table 3). The 3-year DFS rates (62 % vs 63 %; *P* = 0.5) and OS rates (83 % vs 82 %; *P* = 0.8) were similar [73]. Subsequently, the NSABP B-23 trial showed no difference in outcomes in patients who were treated with CMF or AC in patients with node-negative disease [74]. The EBCTCG meta-analysis found that comparison of CMF with AC yielded similar results for breast cancer mortality (HR, 0.98; *P* = 0.67) [16]. Other studies have found no advantage to administration of six compared to four cycles of AC, and superiority for AC compared with single agent paclitaxel given every 2 or 3 weeks [75].

#### 5-Flourouracil, epirubicin (50 mg/m^2^), and cyclophosphamide (FEC50)

The French Adjuvant Study Group (FASG) compared epirubicin-based chemoendocrine therapy with tamoxifen alone in 457 postmenopausal women with ER-positive breast cancer and 1–3 positive nodes enrolled in two trials (FASG 2 and 7) [76]. The chemotherapy regimen was FEC50 (5-fluorouracil 500 mg/m^2^, epirubicin 50 mg/m^2^, cyclophosphamide 500 mg/m^2^), which was given every 3 weeks for six cycles concurrently with tamoxifen. The 9-year DFS rates were 72 % with tamoxifen and 84 % with FEC50-tamoxifen (HR, 0.46; *P* = 0.0008). The 9-year OS rates were 78 % and 86 %, respectively (*P* = 0.11). In the multivariate model, there was a trend in favor of chemoendocrine therapy for OS (HR, 0.65; *P* = 0.07).

### Second generation chemotherapy regimens

#### 5-Flourouracil, epirubicin (100 mg/m^2^), and cyclophosphamide (FEC100)

After randomized trials demonstrated a dose–response relationship for epirubicin in metastatic breast cancer [58, 77], the FASG05 compared adjuvant fluorouracil (500 mg/m^2^) and cyclophosphamide (500 mg/m^2^) with epirubicin given at 50 mg/m^2^ (FEC50) or 100 mg/m^2^ (FEC100) every 21 days for six cycles (FEC50) [78]. After 5 years of follow-up, FEC100 showed improved DFS (HR, 0.63; *P* = 0.02) and OS (HR, 0.45; *P* = 0.005) compared to FEC50 [78] (Table 3).

#### Cyclophosphamide, doxorubicin, and 5-fluorouracil (CAF or FAC)

CAF is an acronym that is used to describe regimens in which cyclophosphamide is administered orally for 14 days and doxorubicin and 5-fluorouracil are given on days 1 and 8 every 28 days for six cycles, whereas FAC is an acronym used to describe a regimen in which all of these agents are given IV every 3 weeks for six cycles. The SWOG-8814/INT-0100 trial randomized postmenopausal women with hormone-receptor positive, node-positive breast cancer to CAF plus tamoxifen versus tamoxifen alone. DFS was superior for CAF plus tamoxifen (HR, 0.76; *P* = 0.002), but OS was only marginally improved (HR, 0.83; *P* = 0.057) [23]. The EBCTCG meta-analysis found that breast cancer mortality rates were reduced more with FAC for six cycles (HR, 0.64; *P* <0.0001) than AC for four cycles (HR, 0.78; *P* = 0.01) or CMF for six cycles (risk ratio, 0.76; *P* <0.0001) [16], and that FAC or FEC combinations were more effective in reducing breast cancer mortality compared to CMF (risk ratio, 0.78; *P* = 0.0004) [16].

#### Sequential doxorubicin/cyclophosphamide followed by paclitaxel (AC-T)

Concurrent administration of paclitaxel and doxorubicin, two of the most active cytotoxic agents for metastatic breast cancer, was associated with substantial activity, but led to prohibitive cardiotoxicity due to a pharmacokinetic interaction resulting in greater doxorubicin exposure [79]. In addition, mathematical modeling predicted that sequential administration of cytotoxic agents at their optimal doses would result in more effective antitumor activity than their concurrent administration [80, 81]. Therefore, two phase 3 trials evaluated sequential administration of paclitaxel after anthracycline-containing therapy (Table 3). CALGB 9344 employed a 2 × 2 factorial design evaluating escalating doses of doxorubicin (60, 75, or 90 mg/m^2^) in combination with cyclophosphamide (600 mg/m^2^) every 21 days for four cycles, given alone, or followed sequentially by four cycles of paclitaxel (175 mg/m^2^ IV every 21 days) in 3,121 patients with node-positive breast cancer. After a median follow-up of 69 months, the addition of paclitaxel was associated with improved DFS (HR, 0.83; *P* = 0.0023) and OS (HR, 0.82; *P* = 0.006). Escalation of doxorubicin dose had no impact on outcomes [82]. In the NSABP B-28 trial, after a median follow-up of 65 months, the sequential addition of four cycles of paclitaxel (225 mg/m^2^) to four cycles of AC in 3,060 patients with node-positive breast cancer improved DFS (HR, 0.83; *P* = 0.006) but not OS (HR, 0.93; *P* = 0.46) [83]. Endocrine therapy with tamoxifen was given after chemotherapy in the C9344 trial in patients with ER-positive disease, whereas it was given concurrently with chemotherapy in the B28 trial for patients 50 years of age or older, and those younger than 50 with ER- and/or PR-positive tumors. Other studies have suggested greater benefit from adjuvant chemotherapy in postmenopausal women with ER-positive disease when tamoxifen is initiated sequentially following completion of chemotherapy [23]. The results of the C9344 study supported FDA approval of adjuvant paclitaxel in the US.

#### Sequential epirubicin followed by CMF

The National Epirubicin Adjuvant Trial (NEAT) and BR9601 trials evaluated the efficacy of epirubicin followed by CMF. The BR9601 trial used a modified CMF regimen (cyclophosphamide 750 mg/m^2^, methotrexate 50 mg/m^2^, and 5-flouorouracil 600 mg/m^2^ on day 1 every 3 weeks) whereas the NEAT trial used classic CMF. Both trials compared four cycles of epirubicin 100 mg/m^2^ every 3 weeks followed by four cycles of CMF (epirubicin-CMF) versus CMF alone (six cycles in NEAT, eight cycles in BR9601). Combined analysis included 2,391 patients. After a median follow-up of 48 months, epirubicin-CMF group was associated with improved DFS (HR, 0.69; *P* <0.001) and OS (HR, 0.67; *P* <0.001) compared to CMF alone [84].

#### Docetaxel plus cyclophosphamide

The US Oncology Research phase III trial evaluated 1,016 patients with operable breast cancer who were assigned to four 3-week cycles of AC (doxorubicin 60 mg/m^2^ and cyclophosphamide 600 mg/m^2^) or DC (docetaxel 75 mg/m^2^ and cyclophosphamide 600 mg/m^2^) [85] (Table 3). After a median follow-up of 84 months, DC was associated with significantly improved DFS (HR, 0.74; *P* = 0.033) and OS (HR, 0.69; *P* = 0.032).

### Third generation chemotherapy regimens

#### Docetaxel, doxorubicin, and cyclophosphamide (DAC)

Unlike paclitaxel, docetaxel does not have a major pharmacokinetic interaction with doxorubicin, and does not increase doxorubicin-related cardiotoxicity when given concurrently [86–88]. Two studies compared a combination of docetaxel, doxorubicin, and cyclophosphamide (DAC) with FAC (Table 3). The Breast Cancer International Research Group 0001 trial compared six cycles DAC (docetaxel 75 mg/m^2^, doxorubicin 50 mg/m^2^, and cyclophosphamide 500 mg/m^2^) with FAC (5-fluorouracil 500 mg/m^2^, doxorubicin 50 mg/m^2^, and cyclophosphamide 500 mg/m^2^) every 3 weeks as adjuvant treatment for 1,491 women with operable node-positive breast cancer [89, 90]. After a median follow-up of 124 months, there were improvements in DFS (HR, 0.80; *P* = 0.0043) and OS (HR, 0.74; *P* = 0.002). The benefit in DFS was irrespective of nodal, hormone receptor, and HER2 status. The GEICAM 9805 trial compared six cycles of DAC with FAC in 1,060 patients with node-negative breast cancer [91]. After a median follow-up of 77 months, there was a significant improvement in DFS (HR, 0.68; *P* = 0.01) and a trend toward improved OS (HR, 0.76; 95 % CI, 0.45 to 1.26; *P* = 0.29) favoring DAC. In both trials, DAC was associated with considerably more toxicity, including febrile neutropenia.

#### Sequential FEC-taxane therapy

Although it was clear that results improved when taxanes were added sequentially following anthracyclines, it was unclear as to whether this improvement was specifically due to the sequential addition of a taxane, or due to more prolonged duration of adjuvant chemotherapy administration. Three trials described herein directly addressed this question by comparing an anthracycline-containing regimen and a sequential anthracycline-taxane regimen in which the treatment arms had a comparable duration (Table 3). The PACS01 trial evaluated six 3-week cycles of FEC (5-fluorouracil 500 mg/m^2^, epirubicin 100 mg/m^2^, and cyclophosphamide 500 mg/m^2^) with three cycles of FEC followed by docetaxel (100 mg/m^2^ every 3 weeks) for three cycles in 1,999 patients with node-positive breast cancer [92]. After a median follow-up of 93 months, the sequential taxane arm was associated with improved DFS (HR, 0.85; *P* = 0.036) and OS (HR, 0.75; *P* = 0.007) [93]. The UK TACT study randomized 4,162 women with node-positive or high-risk node-negative breast cancer to four cycles of FEC followed by four cycles of docetaxel (100 mg/m^2^ every 3 weeks) versus a control regimen consisting of physician’s choice of eight cycles of FEC (5-fluorouracil 600 mg/m^2^, epirubicin 60 mg/m^2^, and cyclophosphamide 600 mg/m^2^) or epirubicin for four cycles followed by four cycles of CMF. After a median follow-up of 62 months, there was no significant difference in outcome between the arms [94]. In the GEICAM 9906 trial, 1,246 patients with node-positive breast cancer patients were randomized to six cycles of FEC (5-fluorouracil 600 mg/m^2^, epirubicin 90 mg/m^2^, and cyclophosphamide 600 mg/m^2^) or four cycles of FEC followed by eight weekly doses paclitaxel (100 mg/m^2^ per week). After a median of 66 months, the sequential paclitaxel arm was associated with a reduced risk of recurrence (HR, 0.77; *P* = 0.022) and trend toward a lower risk of death (HR, 0.78; *P* = 0.110) [95].

The specific benefit of sequential addition of a taxane was also addressed in the 2012 EBCTCG meta-analysis [16]. In trials adding four separate cycles of a taxane to a fixed anthracycline-based control regimen and extending treatment duration, breast cancer mortality was reduced (HR, 0.86; *P* = 0.0005). However, in trials with four such extra cycles of a taxane counterbalanced in controls by extra cycles of other cytotoxic drugs, roughly doubling non-taxane dosage, there was no significant difference in breast cancer mortality (HR, 0.94; *P* = 0.33). Although these results would suggest similar benefits for a 24- compared to a 12-week adjuvant cytotoxic regimen irrespective of which agents are used, the sequential approach may minimize the delayed effects of anthracyclines whose risk increases with greater cumulative dose.

#### Dose dense sequential doxorubicin/cyclophosphamide-paclitaxel (AC-T)

The concept of dose density has been also evaluated in some adjuvant therapies for breast cancer. Dose density refers to administering the same therapeutic regimen (without changing actually doses given) at more frequent intervals, with the goal of decreasing the time for cancer cells to recover in between chemotherapy cycles [80, 96]. The C9741 assessed the impact of dose density (2 week vs 3 week) and treatment sequence (concurrent vs sequential) in patients with operable breast cancer by randomizing 2,005 patients to four different treatment arms using a 2 × 2 factorial design to: (1) concurrent AC-T (paclitaxel) versus sequential A-C-T and (2) every 3 weeks versus a dose-dense regimen every 2 weeks plus filgrastim [97] (Table 3). At 36-month follow-up, the dose-dense regimen improved the primary end-point DFS (HR, 0.74; *P* = 0.01) and OS (HR, 0.69; *P* = 0.013). There was no difference in either DFS or OS between the concurrent and sequential schedules [97]. Updated results after a median 6.5 years of follow-up continue to favor dose-dense chemotherapy in DFS (HR, 0.80; *P* = 0.01) and OS (HR, 0.85; *P* = 0.04) [98]. The dose-dense schedule was associated with improved DFS (HR, 0.76; *P* =0.01) and OS (HR, 0.79; *P* = 0.04) in ER-negative disease but not ER-positive disease.

A systemic review and meta-analysis identified 10 trials that met the inclusion criteria for evaluating the effect of dose-dense chemotherapy scheduling [99]. Three trials, enrolling 3,337 patients, compared dose-dense chemotherapy with a conventional chemotherapy schedule (similar agents). Patients who received dose-dense chemotherapy had improved OS (HR, 0.84; *P* = 0.03) and DFS (HR, 0.83; *P* = 0.005) compared with those who received the conventional schedule, although no benefit was observed in patients with hormone receptor-positive tumors. Seven trials, enrolling 8,652 patients, compared dose-dense chemotherapy with regimens that use standard intervals but with different agents and/or dosages in the treatment arms. Similar results were obtained for these trials with respect to OS (HR, 0.85; *P* = 0.01) and DFS (HR, 0.81; *P* <0.001). The rate of non-hematological adverse events was higher in the dose-dense chemotherapy arms than in the conventional chemotherapy arms.

#### Sequential AC-weekly paclitaxel or every 3 week docetaxel

The ECOG E1199 trial was designed to identify the optimal taxane and schedule. This trial enrolled 4,954 patients with stage II–III breast cancer who received standard AC followed sequentially by taxane therapy using a 2 × 2 factorial design. The study found no difference in the primary comparisons of taxane (paclitaxel vs docetaxel) and schedule (every 3 weeks vs weekly); other pre-specified analyses included a comparison of the standard every 3 week paclitaxel arm (175 mg/m^2^) for four cycles (P3 control arm) with weekly paclitaxel (80 mg/m^2^) for 12 weeks (P1 arm), docetaxel (100 mg/m^2^) every 3 weeks for four cycles (D3 arm), or weekly docetaxel (35 mg/m^2^) for 12 weeks (D1 arm) [100]. After a median follow-up of 5.3 years, the P1 arm was associated with improved DFS (HR, 0.73; *P* = 0.006) and OS (HR, 0.68; *P* = 0.01) compared with the P3 arm. Although improved DFS was also observed for the D3 arm (HR, 0.77; *P* = 0.02) without a survival benefit, it was associated with substantially more toxicity than the P1 arm. In an updated analysis after a median follow-up of 12.1 years, DFS was significantly improved and OS marginally improved for both the P1 arm (HR, 0.84; *P* = 0.011 and HR, 0.87; *P* = 0.09, respectively) and D3 arm (HR, 0.79; *P* = 0.001 and HR, 0.86; *P* = 0.054, respectively). Although weekly paclitaxel improved DFS and OS (HR, 0.69; *P* = 0.010 and HR, 0.69; *P* = 0.019, respectively) in triple negative breast cancer, no experimental arm improved OS for hormone receptor-positive, HER2 non-overexpressing breast cancer [101]. Another trial found no difference in outcomes comparing weekly paclitaxel (80 mg/m^2^ for 12 doses) with biweekly paclitaxel given at a higher dose (175 mg/m^2^ for six doses) given sequentially after AC, although there was more toxicity with biweekly higher dose paclitaxel schedule [102].

#### Sequential versus concurrent taxane administration

The NSABP B30 trial addressed the question of whether docetaxel is best given concurrently with or sequentially following doxorubicin [103]. The study included 5,351 patients with node-positive breast cancer to receive four cycles of AC followed by four cycles of docetaxel (sequential AC-D), four cycles of doxorubicin and docetaxel (AD), or four cycles of doxorubicin, cyclophosphamide, and docetaxel (concurrent DAC). After a median follow-up of 73 months, DFS was improved in the sequential-AC-D arm compared with the AD (HR, 0.80; *P* = 0.001) and the concurrent DAC arm (HR, 0.83; *P* = 0.01), and OS was likewise improved in the sequential-ACD arm compared with the AD arm (HR, 0.83; *P* = 0.03) and concurrent DAC arm (HR, 0.86; *P* = 0.09).

#### Predicting benefit from chemotherapy

In the EBCTC meta-analyses involving taxane-based or anthracycline-based regimens, proportional reductions in risk of recurrence associated with adjuvant chemotherapy were little affected by age, nodal status, tumor diameter or grade, ER expression, or tamoxifen use, and breast cancer mortality was reduced on average by one-third [16]. Several multiparameter gene expression assays have been shown to provide prognostic information in patients with ER-positive breast cancer [7, 8] and also identify which patients derive greatest benefit from adjuvant chemotherapy [29, 30]. Currently available assays include the Oncotype DX^®^ (Genomic Health, Inc., Redwood City, CA), MammaPrint^®^ (Agendia, Inc. USA, Irvine, CA), Prosigna^®^ (Nanostring Technologies, Seattle, WA), and Breast Cancer Index^SM^ (bioTheranostics, Inc., San Diego, CA). Some of these assays are endorsed by evidence-based guidelines for making clinical decisions regarding the use of adjuvant chemotherapy in specific settings [31]. Nevertheless, the assays may not inform treatment decisions in up to approximately 50 % of those tested [104]. Randomized trials are in progress in order to determine whether chemotherapy may be safely spared in patients with tumors associated with low risk signatures who would otherwise have been advised to receive chemotherapy based on classic clinicopathologic features [105, 106]. For example, in the Trial Assigning Individualized Options for Treatment (TAILORx) (NCT00310180), patients with ER-positive, HER2-negative, axillary node negative disease who meet National Comprehensive Cancer Center Network guidelines for recommending adjuvant chemotherapy are assigned to endocrine therapy alone if the Oncotype DX Recurrence Score (RS) is very low (<11) or chemoendocrine therapy if the RS is in the high or high-intermediate range (>25), and are randomized to chemoendocrine therapy versus endocrine therapy if there is a mid-range RS of 11–25[105]. Likewise, in the clinical trial for treatment of endocrine responsive breast cancer (RxPONDER) (NCT01272037), patients with one to three positive axillary nodes are assigned to chemoendocrine therapy if the RS >25 and randomized to chemoendocrine therapy versus endocrine therapy if the RS is <26 (https://clinicaltrials.gov/ct2/show/NCT01272037). The cut points used in these trials differ from the originally classification of low (RS <18), intermediate (RS18-30), and high (RS >30) in order to minimize the potential for chemotherapy under-treatment [105].

### Tailoring the optimal regimen for individual patients

Factors considered in selecting patients for adjuvant therapy include tumor-specific factors, such as tumor size, axillary node metastasis, and tumor biology (i.e. ER/PR and HER2 expression, multiparameter gene expression assays), and patient specific factors such as age, comorbidities, and patient preference. A risk classification and potential therapeutic options for each risk category is proposed in Table 4. Patients with T1a tumors (1–5 mm) and negative nodes are at very low-risk of recurrence and generally do not require systemic chemotherapy. Patients with intermediate or high-risk disease should receive chemotherapy, whereas those with low-risk disease may be considered for chemotherapy if younger (<50–60 years). Patients with high-risk disease requiring chemotherapy are usually advised to receive an anthracycline and taxane containing regimen (i.e. third generation regimen), whereas those with low or moderate-risk disease may be treated with a taxane-containing regimen without an anthracycline (i.e. second generation regimen). All patients with ER- and/or PR-positive disease should always receive at least a 5-year course of endocrine therapy, usually initiated after chemotherapy is completed if given. Patients with HER2-positive disease should also always receive trastuzumab in combination with chemotherapy. Although data for adjuvant pertuzumab is currently lacking, it is recommended by National Comprehensive Cancer Center Network guidelines as a component of adjuvant therapy [107] for high-risk HER2-positive breast cancer based on improved survival when used in metastatic HER2-postive breast cancer [108], and improved pathologic complete response when used in locally advanced breast cancer [109]. On the other hand, other expert panels do not recommend use of adjuvant pertuzumab until results of the APHINITY trial (NCT01358877) become available [110], an adjuvant trial designed to determine whether adding pertuzumab to adjuvant trastuzumab-chemotherapy regimen improves clinical outcomes.

**Table 4:** Commonly recommended adjuvant chemotherapy regimens

Recurrence risk category and definition	Recommended regimens: ER-positive, HER2-negative	Recommended regimens: ER/PR-negative, HER2-negative	Recommended regimens: HER2-positive
Very low risk			
• Node-Neg, T1a	No chemotherapy	No chemotherapy	No chemotherapy
Low risk			
• Node-Neg, T1b	Consider second generation chemotherapy regimen if RS is high	Consider second generation chemotherapy regimen	Consider weekly paclitaxel + H
• Node-Neg, T1c,	Second generation chemotherapy regimen if RS is high (or consider if intermediate)	Second generation chemotherapy regimen	Weekly paclitaxel + H or TCH
Moderate risk			
• Node-Neg, T2	Second or third generation chemotherapy regimen if RS intermediate-high	Third generation chemotherapy regimen	AC-T + H or TCH +/− P
High risk			
• 1+ Pos Nodes or T3	Third generation chemotherapy regimen if RS intermediate-high (or 4+ positive nodes irrespective of RS)	Third generation chemotherapy regimen	AC-T + H or TCH+/−P

TCH, Docetaxel, carboplatin, trastuzumab; T, Paclitaxel; AC, Doxorubicin, cyclophosphamide; H, Trastuzumab; P, Pertuzumab; Neg, Negative; Pos, Positive; RS, Recurrence score.

## Conclusions

Localized and regionally advanced breast cancer is a potentially curative disease with local therapy alone, and adjuvant systemic chemotherapy, endocrine therapy, and anti-HER2 directed therapy substantially reduce the risk of distant recurrence and breast cancer mortality. Acute reversible effects associated with chemotherapy include alopecia, nausea, vomiting, fatigue, and myelosuppression, whereas long-term potentially irreversible effects include cardiomyopathy, acute leukemia, and neuropathy [111]. The choice of chemotherapy regimen may be individualized based upon disease-specific factors such as the underlying risk of recurrence and the projected relative and absolute benefits from chemotherapy, as well as patient-specific factors such as age, comorbidities, and risk tolerance. Decision aids may be helpful in allowing patients and caregivers to make more informed decisions about the potential benefits of adjuvant chemotherapy, and multiparameter gene expression assays may allow more accurate estimates of the potential benefits of such therapy. The TAILORx, MINDACT, RxPONDER, and OPTIMA trials are evaluating the incorporation of multiparameter gene expression assays into clinical decision making to tailor adjuvant treatment among patients with breast cancer. Improvements in adjuvant cytotoxic regimens have contributed to declining breast cancer mortality rates and clinical trials are underway that may serve to identify subgroups deriving the greatest benefits from such therapy.

## Box 1. Milestones in the adjuvant therapy of breast cancer

Chemotherapy

- Alkylating agents (thiotepa, L-phenylalinine mustard) given after surgery decrease recurrence rates [20, 21]
- Adjuvant polychemotherapy “CMF” regimen substantially reduces risk of recurrence [22] and improves survival [12, 25]
- National Institute of Health consensus panel recommends that adjuvant polychemotherapy be recommended to the majority of women with localized breast cancer regardless of lymph node, menopausal, or hormone receptor status [27]
- Anthracyclines and taxanes integrated into adjuvant chemotherapy regimens produce additional survival gains [16]
- Multiparameter gene expression assays identify subsets of patients with ER-positive disease who derive greatest benefit from adjuvant chemotherapy [29, 30] and are incorporated into evidence-based guidelines [31]

Endocrine therapy

- Adjuvant tamoxifen reduces recurrence [32] and improve survival [12, 33]
- Five years of adjuvant tamoxifen therapy more effective than shorter durations [13]
- The proportional benefits of endocrine therapy are similar irrespective of nodal metastasis and that the benefits are seen only in patients with ER-positive tumors [15]
- Aromatase inhibitors are more effective than tamoxifen in postmenopausal women [34, 35]
- Extended adjuvant therapy for up to 10 years more effective than 5 years of therapy, including sequential tamoxifen followed by aromatase inhibitor [36], or tamoxifen for up to 10 years [37]
- Ovarian suppression plus an aromatase inhibitor was shown to be more effective than tamoxifen in premenopausal women at high risk for recurrence [38, 39]

Anti-HER2 therapy

- Adjuvant trastuzumab reduces the risk of recurrence when added to adjuvant chemotherapy, given either concurrently or sequentially, in patients with HER2 overexpressing node-positive or high-risk node-negative breast cancer [43]
- One year of trastuzumab was more effective than 6 months [46], 2 years of therapy was no more effective than 1 year [47]
- Addition of the HER2 tyrosine kinase inhibitor did not improve outcomes when added to trastuzumab [48]

## Abbreviations

AC: Doxorubicin and cyclophosphamide
CAF: Cyclophosphamide, doxorubicin, and 5-flourouracil
CMF: Cyclophosphamide, methotrexate, and 5-fluorouracil
DAC: Docetaxel, doxorubicin, and cyclophosphamide
DC: Docetaxel and cyclophosphamide
DFS: Disease-free survival
EBCTCG: Early Breast Cancer Trialists’ Collaborative Group
ER: Estrogen receptor
FASG: French Adjuvant Study Group
FDA: United States Food and Drug Administration
FEC50: 5-Fluorouracil, epirubicin, and cyclophosphamide
HER2: Human epidermal growth factor receptor 2
HR: Hazard ratio
NEAT: National Epirubicin Adjuvant Trial
NSABP: National Surgical Adjuvant Breast and Bowel Project
OS: Overall survival
PR: Progesterone receptor
RS: Recurrence score
